# Analysis of the antioxidant activity of toons sinensis extract and their biological effects on broilers

**DOI:** 10.3389/fvets.2023.1337291

**Published:** 2024-01-08

**Authors:** Xiangmin Zhao, Baolong Du, Minyan Wan, Jinlu Li, Shizhen Qin, Fang Nian, Defu Tang

**Affiliations:** ^1^College of Animal Science and Technology, Gansu Agricultural University, Lanzhou, China; ^2^Yizhou District Animal Disease Prevention and Control Center, Hami, China; ^3^College of Science, Gansu Agricultural University, Lanzhou, China

**Keywords:** broilers, Toona sinensis extract, growth performance, meat quality, serum antioxidant, intestinal microbes

## Abstract

Plant extracts are rich in a variety of nutrients and contain a large number of bioactive compounds, and compared with traditional feed additives, they have advantages such as wide sources, natural safety and rich nutrition. This study employed *in vitro* antioxidant and animal experiments to comprehensively evaluate the use of Toona sinensis extract (TSE) in broiler production. 508 1-day-old Cobb 500 broilers were randomly assigned to the 7 experimental groups with 6 replications and 12 birds/replicate. Two groups received Vitamin C (VC) 300 g/t and Vitamin E 500 g/t, and five dose groups of TSE received 0, 300, 600, 900, and 1,200 g/t of TSE in their feed. The study spanned 42 days, with a starter phase (1–21 days) and a finisher phase (22–42 days). The results showed that compared to ascorbic acid, TSE had the scavenging ability of 2,2-Diphenyl-1-picrylhydrazyl and hydroxyl radical, with IC_50_ values of 0.6658 mg/mL and 33.1298 mg/mL, respectively. Compared to TSE _0_ group, broilers fed with 1,200 g/t TSE showed significant weight gain during the starter phase and increased the feed-to-weight gain ratio during both the starter and finisher phases. Additionally, broilers receiving 1,200 g/t TSE had enhanced dry matter and organic matter utilization. Concerning meat quality, broilers in the 1,200 g/t TSE group demonstrated increased cooked meat yield, and pH value, as well as higher antioxidant capacity (T-AOC), dismutase (SOD), and glutathione peroxidase (GSH-PX) in serum. In addition, there was no significant difference in ileal microflora due to TSE supplementation. In summary, this study confirms the positive impact of a dietary inclusion of 1,200 g/t TSE on broiler growth, meat quality, and serum antioxidants.

## Introduction

With the development of the economy and the improvement of living standards, people’s demand for animal products continues to grow, which has promoted the industrial development of animal husbandry. However, backward production capacity will gradually be eliminated by the tightening of national policies. In the context of enhanced environmental protection policies and government regulation, antibiotics play a key role in animal growth and development, improving feed conversion efficiency, and preventing infections (1, 2). However, the largescale use of antibiotics has led to growing concerns about drug resistance and human health risks (3). In recent years, many countries have restricted or even banned the use of antibiotics in animal feed (4). Since July 1, 2020, the production of commercial feed containing growth-promoting drugs and other feed additives has been banned in China. Therefore, reducing or replacing the use of antibiotics has become an urgent issue for the breeding industry. Recent studies have shown that active substances such as flavonoids and polyphenols et al. in plant extracts can enhance the innate immunity of animals, promote growth and development, reduce oxidative stress, maintain intestinal integrity, promote the growth of beneficial bacteria, and reduce intestinal infections (5).

Toona Sinensis (A. Juss.) Roem (TSR), an indigenous Meliaceae family plant and a deciduous woody species, is native to East and Southeast Asia (6). It has been cultivated for over 2,000 years in China, Malaysia, and other parts of Asia as a popular seasonal vegetable (7). In addition, TSR has been utilized as a Chinese patent medicine in Traditional Chinese Medicine (TCM) for various ailments. For instance, TSR bark is used for treatments of dysentery, enteritis, and urinary infections; leaves for dysentery; seeds for chronic gastritis; and the roots are used for heat-eliminating, detoxifying, and astringent properties (8, 9). Previous studies have validated the therapeutic effects of TSR, which are primarily attributed to its flavonoids, phenols, terpenoids, and other bioactive sub-stances that contribute to metabolic regulation and growth and development (10, 11), These bioactive substances not only promote the absorption and metabolism of nutrients but also enhance the quality of livestock products (meat quality, dairy quality, etc.) (12, 13). Furthermore, modern medical research has demonstrated various medicinal properties of TSR. It can regulate lipid metabolism, alleviate hyperglycemia, enhance immunity, delay liver fibrosis, regulate microcirculation, affect blood pressure, combat oxidative stress, induce cancer cell apoptosis, inhibit sarcoma growth, etc. (14–16). Despite its established role in TCM, food, and compounds, there has been limited literature on its application in poultry and animal production, particularly in broiler production. Based on previous findings, we hypothesize that TSR extract (TSE) could serve as a novel feed additive to enhance animal growth performance in specific aspects. The aim of the current study was to evaluate the TSE *in vitro* antioxidant activity, and its impact of supplementing broiler chickens with varying amounts of these extracts on growth performance, meat quality, serum antioxidants, and intestinal microbial diversity were also investigated.

## Materials and methods

### Chemicals and reagents

The Toona Sinensis Extract (TSE) used in this experiment was supplied by Personalbio Technology Co., Ltd. as a powder preparation with an effective active substance content of 10%. VC (Vitamin C)-standard products, 2,2-Diphenyl-1-picrylhydrazyl (DPPH) reagents, and salicylic acid were purchased from Jiangsu Enzyme Industry Co., Ltd.

### *In vitro* antioxidant activity assay

The DPPH radical scavenging ability was assessed following the methods of Hu et al. (17). Hydroxyl radical scavenging assays were conducted following the procedure of Li et al. (18). Ascorbic acid (VC) was used as a positive control to evaluate the DPPH and hydroxyl radical scavenging activities of the TSE comparatively.

### Broiler feeding management and experimental design

We sourced 504 healthy, 1-day-old Cobb500 male white-feathered broilers (50 ~ 55 g) from Shaanxi Baoji Hualong Animal Husbandry Co., Ltd., China. These birds were randomly allocated into seven distinct groups. Each group consisted of 6 replicates, and each replicate had 12 birds. The dietary interventions for seven groups involved basic feeds supplemented with VC 300 g/t, Vitamin E (VE) 500 g/t, and five dose groups of TSE, including 0, 300, 600, 900, and 1,200 g/t, respectively (The influence factors of animal tests are complex, and double positive controls are needed to confirm the expected effect and exclude accidental factors, so as to improve the credibility and scientific results). In addition, the grower diet contained 0.4% titanium dioxide per kg feed as an indigestible marker to allow for the determination of nutrient digestibility. The precise formulation and nutritional constituents of these feeds are listed in Table 1. The study spanned 42 days with two distinct phases: the “starter” phase (1–21 days) followed by the “finisher” phase (22–42 days). The broilers were randomly allocated, according to similar body weight, to individual wire cages (160 × 70 cm^2^) with raised-wire floors and automatic nipple drinkers. Throughout the study duration, the broilers resided in a controlled environment with free access to feed and water. For the first 3 days, the temperature was set at 36–38°C, and thereafter, we systematically reduced it by 5°C every week to 24°C. This environment ensured 23 h of consistent lighting, interspersed with a one-hour dark period.

**Table 1:** Ingredient composition of basal diets (air-dry basis, %).

Ingredients	Starter (1–21 d)	Finisher (22–42 d)
Maize	56.00	57.70
Corn oil	2.60	4.70
Soya bean meal	21.43	18.00
Cottonseed meal	5.00	5.00
Rapeseed meal	5.00	5.00
Distillers dried grains with solubles	5.00	5.00
Limestone	1.20	1.10
Dicalcium phosphate	1.70	1.20
premix^1^	0.50	0.50
Nacl	0.35	0.35
DL-Methionine	0.28	0.16
L-Lysine	0.50	0.48
Sodium bicarbonate	0.24	0.24
Choline chloride	0.20	0.20
TiO_2_	0	0.40
Total	100.00	100.00
ME. Kcal/kg	2950.00	3100.00
CP, %	20.00	18.00
Ca, %	1.00	0.80
Available P, %	0.46	0.35
Met, %	0.54	0.40
Lys, %	1.15	1.05

^1^Provided per kg of diet: Vitamin A, 15000 IU; Vitamin D3, 5,100 IU; Vitamin E, 25.5 mg; Vitamin. K, 2.5 mg; Vitamin B1, 3.6 mg; Vitamin B2, 6.9 mg; Vitamin B6, 4.5 mg; Vitamin B12, 0.036 mg; Nicotinic, 66.3 mg; Calcium pantothenate, 18 mg; Biotin, 0.18 mg; Folic acid, 1.4 mg, Fe 55 mg, Zn 60 mg, Mn 60 mg, Se 0.2 mg, I 0.3 mg, Cu 12.8 mg.

### Sample collection and Indicator measurement

#### Growth performance

Any incidences of animal mortality were noted for all cages daily. On the 21st and 42nd mornings, after ensuring a 12-h fasting period, both the average feed intake (AFI) and the average weight gain (AWG) were systematically assessed. To ensure data accuracy, parameters such as the feed-to-weight gain (F/G) ratio and related metrics were used for adjustments to account for any losses due to mortality.

#### Apparent total tract digestibility

During the 38 to 41 days, each replicate cage collects excrement and removes impurities from it (5 mL sulfuric acid was added to the collection bowl to prevent nitrogen release before collection), and stored at −20°C. At the end of the collection period, three days of fecal samples were thawed and combined, then dried at 65°C for 72 h for determination dry matter (DM), organic matter (OM), nitrogen (N), and metabolizable energy (ME). The TiO_2_ assay was conducted as described by Morgan et al. (19). DM, OM, and N levels were estimated using the methods outlined by the AOAC (20). The ME of samples was determined using a calorimeter (C2000, IKAWERKE Co., Ltd., Staufen, Germany).

#### Meat quality analysis

Meat color (a* redness, b* yellowness, and L* brightness), drip loss, and pH value were determined as described by Jin et al. (21). After 45 min of slaughter, the meat color inside the breast and thigh muscles was measured twice (rotated 90°) with a 50 mm aperture colorimeter (CR410, Minolta, Japan) to calculate the average value. Subsequently, the meat sample was stored at 4°C for 24 h for a repeat measure of the meat color. The pH value of the breast and thigh meat was measured 45 min and 24 h after slaughter using a pH electrode meter (HI99161, Hanna, Italy). Drip loss was determined as described by Jin et al. (21). Briefly, muscle blocks (2 cm thick, 3 cm wide, and 5 cm long) were cut from the same position of the pectoralis and thigh muscles, weighed, put into zipper bags, and hung freely. After storage at 4°C for 24 h, the pieces were reweighed to obtain the final weight. This final weight was used to assess drip loss.

$\begin{array}{l} \mathrm{Drip}\;\mathrm{loss}\;\left(\%\right)=\left(\mathrm{Initial}\;\mathrm{weight}:\mathrm{Final}\;\mathrm{weight}\right)/\\ \mathrm{Initial}\;\mathrm{weight}\times 100 \end{array}$

The meat shear force was determined as described by Jin et al. (21). Briefly, meat samples were retrieved from 0–4°C storage and then put in a constant-temperature water bath set at 80°C. The sample was heated until the core temperature reached 70°C, then cooled down to 0–4°C. Using a circular sampler with a diameter of 1.27 cm, meat samples were drilled and cut parallel to the direction of the muscle fibers. The length of the sampling hole should not be less than 2.5 cm, and the sampling position should be at least 5 mm from the edge of the sample. The distance between the edges of two adjacent samples should be no less than 5 mm. Meat shear force was immediately measured with a digital muscle pressure pain tester (C-LM3B, TENOVO, China).

Water loss was determined using the filter paper press method as described by Farouk et al. (22). About 1 g of the meat sample (W_1_) was weighed. After placing 16 layers of qualitative filter paper above and below the meat sample, a nonabsorbent plastic plate was added on both sides. The sample was then placed on the dilatometer platform and subjected to a continuous pressure of 68.66 KPa for 5 min. After the pressure was removed, the meat sample was weighed again (W_2_). The water loss rate was calculated as follows:

$\mathrm{Water}\;\mathrm{loss}\;\mathrm{rate}\;\left(\%\right)=\left(W_{1}:W_{2}\right)/W_{1}\times 100$

The meat cooking rate was determined within 12 h of slaughtering. An appropriate amount of chicken breast meat (W_1_) was put in a steamer (1,500 W) and steamed at 100°C for 45 min. The meat was then taken out and cooled for 30–40 min in a windless room. A filter paper was used to blot the surface moisture, and ultimately the meat was weighed (W_2_) to determine the cooking rate as follows:

$\mathrm{Cooked}\;\mathrm{rate}\;\left(\%\right)=\left(W_{2}:W_{1}\right)\times 100$

#### Antioxidant capacity

At 42 d, one bird from each replicate was randomly selected. Blood samples (5–10 mL) were obtained from the inferior wing vein. The serum samples were collected by centrifugation at 3000 × g at 4°C for 10 min and stored at −80°C for further serum antioxidant indices analysis. The kits provided by Jiangsu Enzyme Industry Co., Ltd. were used to measure the levels of superoxide dismutase (SOD), glutathione peroxidase (GSH-PX), malonaldehyde (MDA), and total antioxidant capacity (T-AOC) following the kits’ instructions and a spectrophotometer.

#### Intestinal microbial diversity

The measurement indicators in this study included effective sequences, optimized sequences, OTUs (operational taxonomic units), species abundance, and community structure. Immediately after the dissection, the ileum was stripped, and the ileal contents were collected in 5 mL cryovials, which were quickly frozen in liquid nitrogen and then stored at −80°C. We performed paired-end (PE) sequencing to determine the intestinal microbial diversity. FLASH software was used for data organization and control, and a small fragment sequence library was constructed for sequencing. Species composition and variability between samples were explored by data filtering and analysis of species annotation curves, abundance distribution curves, alpha diversity, beta diversity, and species distribution. This analysis was aimed at examining the composition and differences of species in a sample. The sequencing of the gut microbiota was performed by Biomicr Biotechnology Co., Ltd., China.

#### Data statistics and processing

All data were collated in Excel 2007 and then processed in SPSS 22.0 with the One-way ANOVA method. When the difference was significant, Duncan’s method was used for multiple comparison tests. *p* ≤ 0.05 was set as the cut-off significance level. A trend was identified when 0.05 < *p* < 0.10. Bioinformatic analysis of microbe-related data was conducted using Qiime, Mothur, and MEGAN4 programs.

## Results

### Antioxidant activity of TSE and ascorbic acid *in vitro*

**Figure 1:**
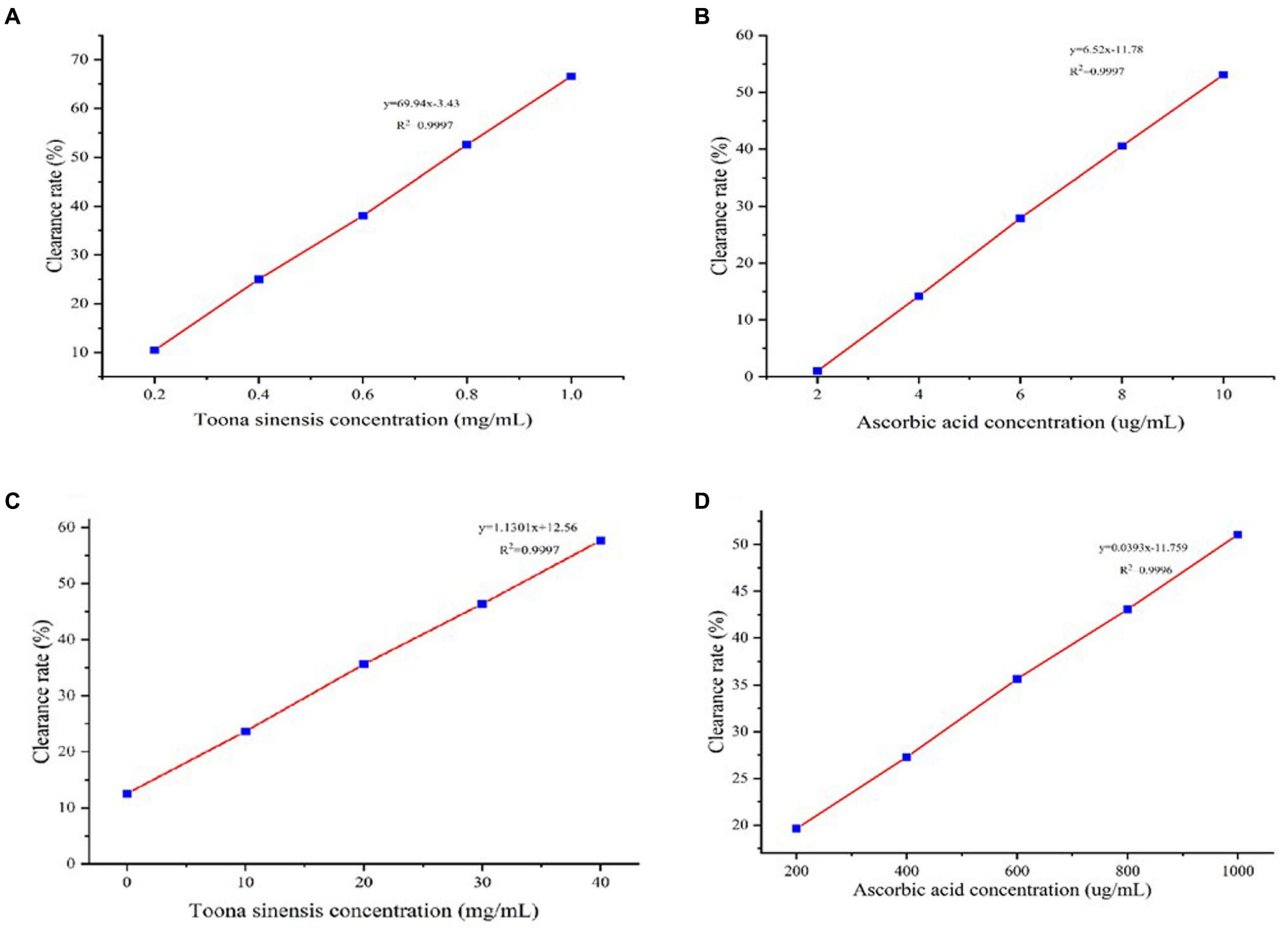
The antioxidant activities of TSE and VC were assessed by measuring their ability to scavenge DPPH and hydroxyl radicals. **(A)** DPPH radical scavenging rate curve of TSE, **(B)** DPPH. radical scavenging rate curve of ascorbic acid, **(C)** Hydroxyl radical scavenging rate curve of TSE, and **(D)** Hydroxyl radical scavenging rate curve of ascorbic acid.

A DPPH free radical assay was performed to assess the antioxidant activity of TSE and ascorbic acid. After their interactions with free radical scavengers, the purple color of the DPPH solution turned lighter; sample absorbance was measured at 517 nm. Figure 1A shows a clear linear relationship (y = 69.94x-3.43, *R*^2^ = 0.9997) between TSE con-centration and DPPH radical scavenging rate, i.e., with the increasing TSE concentration, its ability to scavenge DPPH free radicals increased. The IC_50_ concentration of TSE to achieve 50% clearance of DPPH was 0.6658 mg/mL. We also determined the DPHH-scavenging ability of ascorbic acid as a positive control. As shown in Figure 1B, ascorbic acid also has a linear scavenging rate of DPPH radicals. Based on the regression equation of y = 6.52x-11.78, *R*^2^ = 0.9997, the IC_50_ of ascorbic acid was determined to be 9.47546 μg/mL.

We next determined the hydroxyl radical scavenging activity of TSE and ascorbic acids. The TSE scavenging rate of hydroxyl radicals is shown in Figure 1C. Based on the regression equation of y = 1.301x + 2.56, *R*^2^ = 0.9997, the IC_50_ concentration of TSE to achieve 50% clearance of hydroxyl radicals was 33.1298 mg/mL. Likewise, we deter-mined the hydroxyl radical scavenging activity of ascorbic acid (VC) (Figure 1D). Based on the regression equation of y = 0.0393x-11.759, *R*^2^ = 0.9997, the IC_50_ concentration of ascorbic acid to achieve 50% clearance of hydroxyl radicals was 973.05 μg/mL.

### Effect of TSE on the growth performance of broilers

As shown in Table 2, in the starter stage (1–21 days), there was no significant difference in AFI between treatment groups and control groups (*p* > 0.05), compared with negative control group (TSE _0_) dietary supplementation of VC 300 g/t, VE500 g/t, TSE 300 g/t, 600 g/t, 900 g/t and 1,200 g/t significantly increased AWG (*p* < 0.05), adding TSE 300 g/t and 1,200 g/t significantly increased the AWG (*p* < 0.05), and at the same time significantly reduced the F/G (*p* < 0.05).

**Table 2:** Effect of TSE on the growth performance of broilers.

Item (g/t)	Starter phase (1–21d)			Finisher phase (22–42d)			Total period (1–42d)		
	AFI (g)	AWG (g)	F/G	AFI (g)	AWG (g)	F/G	AFI (g)	AWG (g)	F/G
VC _300_	919.92	649.71^abc^	1.42^b^	2172.26	1151.45	1.89	3082.01	1793.52	1.73^ab^
VE _500_	909.61	645.70^abc^	1.41^b^	2122.73	1110.73	1.99	3132.43	1756.41	1.74^ab^
TSE _0_	911.23	616.81^c^	1.48^a^	2185.86	1082.03	2.01	3120.03	1680.11	1.81^a^
TSE _300_	900.80	667.82^ab^	1.38^bc^	2127.62	1083.32	1.97	3041.67	1749.96	1.72^ab^
TSE _600_	889.76	648.04^abc^	1.37^bc^	2105.86	1090.55	1.90	2992.27	1738.12	1.72^ab^
TSE _900_	888.53	633.42^bc^	1.39^bc^	2217.87	1118.65	1.93	3115.29	1777.34	1.70^ab^
TSE _1200_	909.87	670.26^a^	1.33^c^	2151.63	1095.42	1.90	3052.65	1798.94	1.68^b^
S.E.M	4.93	4.62	0.01	20.56	11.21	0.02	22.07	12.28	0.02
*p* value	0.586	0.026	0.011	0.745	0.180	0.058	0.661	0.106	0.032

AFI, average feed intake. AWG, average weight gain. F/G, feed to weight gain ratio. Values with the same or no letter superscripts within the same row denote no significant difference (*p* > 0.05), while with different letter superscripts indicate significant difference (*p* < 0.05). The same applies to other Tables.

In the finisher stage (22–42 days), compared with the negative control group (TSE _0_), the AFI and F/G of broilers in each treatment were decreased and increased AWG, but not statistically significant (*p* > 0.05).

In the total period (1–42 days), dietary supplementation of VC 300 g/t, VE500 g/t, TSE 300 g/t, 600 g/t, 900 g/t and 1,200 g/t significantly decreased the F/G (*p* < 0.05). Dietary supplementation with 300 g/t VC, TSE 300 g/t, 600 g/t, 900 g/t and 1,200 g/t decreased the AFI. Adding VC 300 g/t, VE500 g/t, TSE 300 g/t, 600 g/t, 900 g/t, 1,200 g/t increased the AWG, but there was no significant difference (*p* > 0.05).

### Nutrient utilization efficiency

As shown in Table 3, compared to other treatments, the broiler chickens fed TSE 900 and TSE 1200 g/t retained the highest amount of DM (*p* < 0.05). The dietary supplementation of the VC, VE and TSE significantly improved the OM utilization rate of broilers (*p* < 0.05), and the best effect was exhibited by the TSE 1200 g/t. Except for the TSE 300 g/t, all other groups exhibited a significant increase in nitrogen metabolism rate (*p* < 0.05), with the TSE 900 g/t demonstrating the highest value (*p* < 0.05). In addition, dietary supplementation of VC, VE and TSE had no significant effect on ME (*p*>0.05).

**Table 3:** Effect of TSE on nutrient utilization of broilers.

Item (g/t)	DM (%)	OM (%)	*N* (%)	ME (%, MJ/kg)
VC _300_	80.33^bc^	83.42^a^	74.36^ab^	13.37
VE _500_	80.54^bc^	80.64^d^	74.48^ab^	13.34
TSE _0_	79.91^c^	80.01^e^	73.51^b^	13.30
TSE _300_	79.52^c^	79.66^d^	73.22^b^	13.16
TSE _600_	81.54^ab^	81.66^c^	74.04^ab^	13.24
TSE _900_	82.15^a^	82.27^b^	75.47^a^	13.16
TSE _1200_	82.67^a^	83.45^a^	74.47^ab^	13.51
S.E.M	0.24	0.45	2.00	0.57
*p* value	<0.001	<0.001	0.048	0.678

Dry matter (DM), organic matter (OM), nitrogen (N), and metabolizable energy (ME).

### Effect of TSE on meat quality

The results of the meat quality analysis are listed in Table 4. Compared with the TSE 0 g/t, the dietary supplementation of VC and VE increased the drip loss of chicken (*p <* 0.05), TSE 300, 600, and 900 g/t effectively reduced the drip loss of chicken, but there was no significant difference (*p* > 0.05). The dietary supplementation with VC, VE, and TSE significantly improved the shear force and cooked yield of breast and thigh muscles (*p <* 0.05); however, we noticed no significant effect on the meat color or pH_45min_ on breast and thigh muscles (*p* > 0.05). Notably, pH_24h_ was significantly increased (*p* < 0.05).

**Table 4:** Effect of TSE on meat quality of broilers.

Items (g/t)	Drip loss rate (%)	Shear force%	Water loss rate%	Cooked yield%	pH		Meat color					
					45 min	24 h	45 min			24 h		
							a	b	L	a	b	L
VC 300	4.44^a^	63.44^a^	70.51	72.66^a^	6.13	5.76^b^	40.32	44.04	62.43	34.83	46.113	55.26
VE 500	4.50^a^	63.90^a^	70.08	68.72^b^	6.04	5.76^b^	38.59	46.50	63.89	36.56	47.43	55.72
TSE 0	3.79^ab^	48.80^b^	71.11	64.10^d^	6.12	5.74^b^	35.22	46.78	64.83	35.53	48.71	57.04
TSE 300	3.62^ab^	63.34^a^	72.64	65.47^cd^	6.29	5.89^a^	38.07	47.40	66.69	33.33	45.51	56.44
TSE 600	3.47^ab^	63.82^a^	73.08	67.09^bcd^	6.28	5.93^a^	36.81	47.62	65.45	32.31	46.01	56.02
TSE 900	3.15^ab^	65.21^a^	73.38	68.46^bc^	6.24	5.90^a^	38.71	47.51	65.21	34.11	46.15	55.14
TSE 1200	3.82^b^	68.44^a^	74.58	67.40^bc^	6.21	5.88^a^	38.74	46.83	67.38	34.34	45.02	56.74
S.E.M	0.13	1.56	0.55	0.51	0.31	0.17	0.45	0.54	0.54	0.43	0.38	0.32
*p* value	0.041	0.022	0.260	<0.001	0.268	0.001	0.070	0.626	0.240	0.156	0.160	0.636

### Effect of TSE on serum antioxidants

The results listed in Table 5 show that the dietary supplementation of antioxidants and TSE significantly increased the serum contents of T-AOC, GSH-PX, and SOD in broilers (*p* < 0.05). The contents of T-AOC, GSH-PX and SOD in serum of broilers were increased with the increase of dietary TSE dose (*p* < 0.05). In addition, the dietary supplementation of antioxidants and TSE significantly reduced the MDA content in broiler serum (*p* < 0.05), and with the increase of TSE addition, MDA content showed a continuous decrease (*p* < 0.05).

**Table 5:** Effect of TSE on antioxidant indices of broilers.

Items (g/t)	T-AOC (U/L)	GSH-PX (U/L)	SOD (U/L)	MDA (U/L)
VC _300_	25.51^a^	234.61^b^	567.62^a^	8.00^e^
VE _500_	24.59^b^	237.36^a^	556.44^b^	8.49^e^
TSE _0_	9.51^f^	126.07^f^	233.58^g^	14.99^a^
TSE _300_	12.59^e^	149.33^e^	356.44^f^	13.34^b^
TSE _600_	16.99^d^	168.58^d^	409.59^e^	11.45^c^
TSE _900_	22.44^c^	194.52^c^	476.55^d^	9.59^d^
TSE _1200_	24.36^b^	237.40^a^	534.15^c^	8.55^e^
S.E.M	1.15	8.18	21.84	0.49
*p* value	<0.001	<0.001	<0.001	<0.001

Total antioxidant capacity (T-AOC), glutathione peroxidase (GSH-PX), superoxide dismutase (SOD), and malonaldehyde (MDA).

### Effect of TSE on the intestinal microflora of broilers

#### Gut microbial raw data processing and sample sequence number statistics

According to the statistical results of production performance of broiler chickens during feeding, AWG and F/G of TSE1200g/t group are more advantageous than those of other treatment groups. In addition, the chickens in this test are commercial generation. Therefore, under the premise of economic benefits, the ileum microbiota of TSE 0 g/t (group C) and TSE 1200 g/t (group T) broilers was further studied.

In this experiment, we processed the PE sequencing data obtained by Hiseq sequencing by splicing (merging) them into sequence tags based on the overlap between PE reads. We also conducted quality control and filtering for the read quality and merge effect. FLASH v1.2.7 software was used to splice samples and obtain the original data, followed by data filtering using the Trimmomatic v0.33 software. The optimized number of sequences was then subjected to chimeric sequence removal by UCHIME v4.2 software. Data in Table 6 show that the effective sequence count for broiler ileum in the group C was 12,787, with 12,683 sequences for subsequent analysis. In the group T, with effective sequence count of the ileum was 12,848, with 12,357 sequences for subsequent analysis.

**Table 6:** Statistical analysis of ileum sequencing samples from broilers.

Sample	Valid sequence	Analysis sequence	Sample	Valid sequence	Analysis sequence
C_1_	13192	12402	T^1^	13897	13585
C_2_	12528	12023	T^2^	13451	13010
C_3_	13720	13414	T^3^	12698	12150
C_4_	11980	11223	T^4^	13933	13569
C_5_	12091	11552	T^5^	11740	10932
C_6_	13213	12683	T^6^	11372	10894
Average	12787	12683	Average	12848	12357

#### Ileal microbial diversity

**Figure 2:**
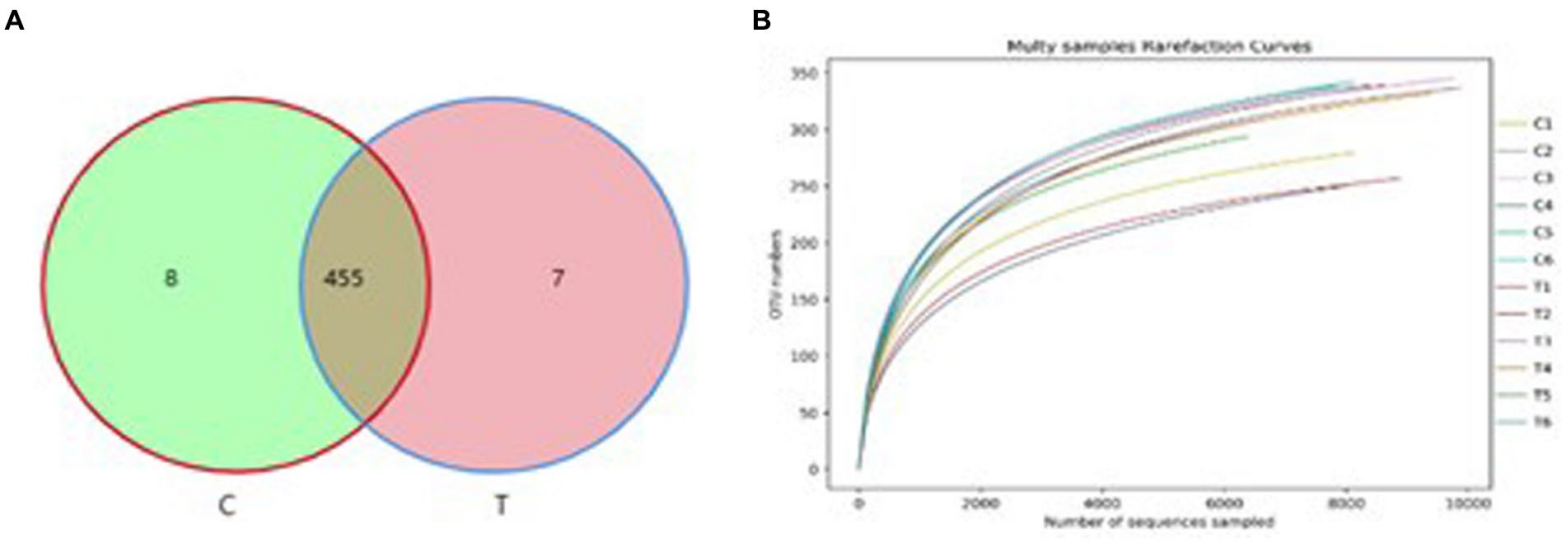
OTU statistical analysis. **(A)** Analysis results of OTU-Venn and **(B)** Dilution curve analysis.

The QLLME 1.8.0 software was utilized for clustering with a similarity threshold set at 97%, resulting in the detection of a total of 470 OTU. Specifically, there were 463 OTU observed for TSE 0 g/t (group C) and 462 OTU for TSE 1200 g/t (group T) (Figure 2A). The dilution curves, which illustrate the species’ diversity and richness in each sample, exhibit a plateau at 8,000 reads, indicating that the sequencing coverage has reached saturation (Figure 2B).

#### Diversity analysis

**Figure 3:**
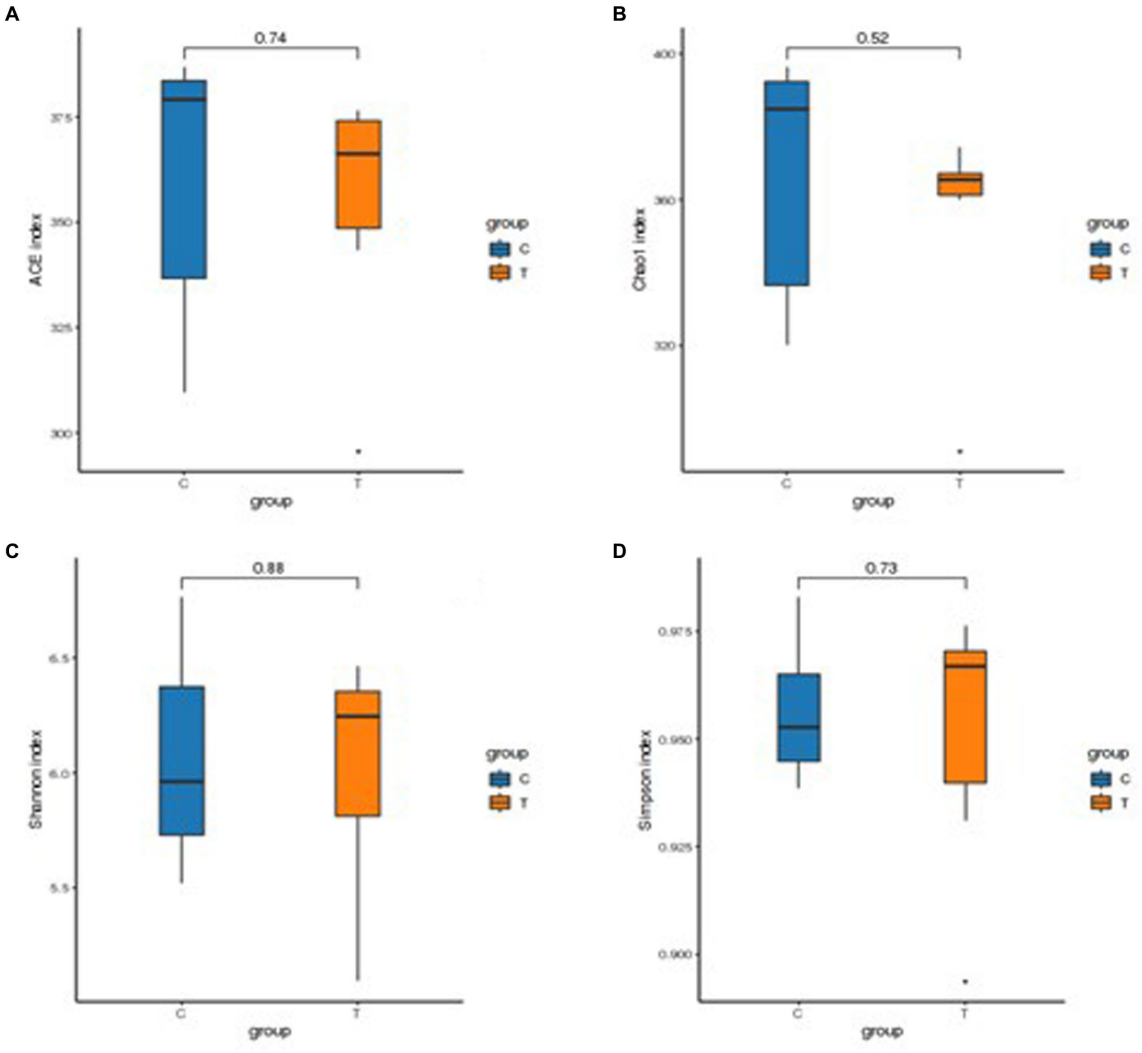
Statistical results of alpha diversity analysis of intestinal flora. **(A)** ACE index, **(B)** Chao 1 index, **(C)** Shannon index, and **(D)** Simpson index.

**Figure 4:**
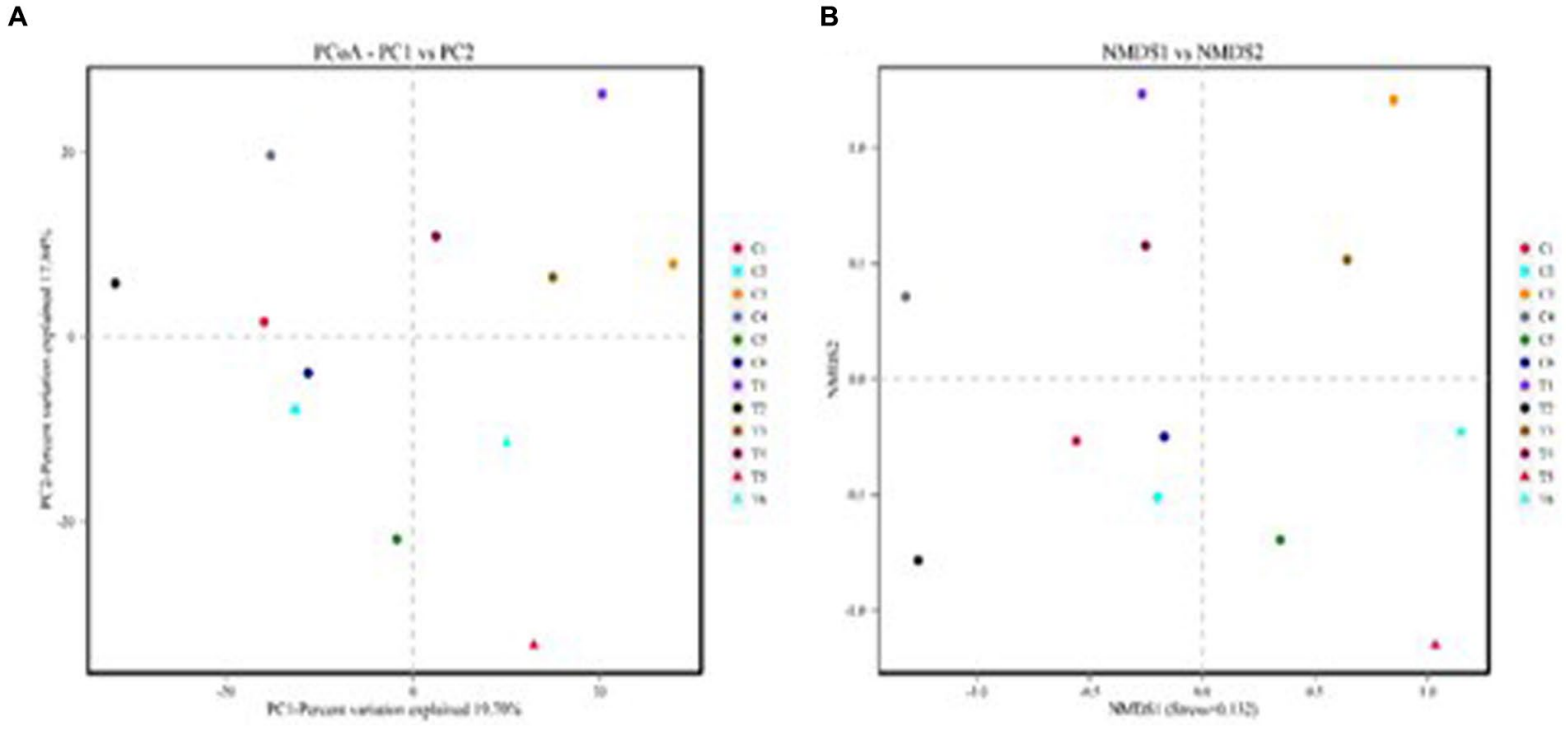
Statistical results of beta diversity analysis of intestinal flora. **(A)** PCoA and **(B)** NMDS.

The results of the alpha-diversity analysis showed that the dietary TSE supple-mentation had no significant effect on the abundance and diversity of ileal microorganisms in broilers (Figure 3). Principal coordinates analysis (PCoA) and non-metric multidimensional scaling (NMDS) are dimensionality reduction techniques that employ a two-dimensional plane to visually represent the dissimilarity between samples. The x and y axes of the PCoA plot corresponded to 19.70% and 17.84% contributions, respectively (Figure 4A). This indicated that the data effectively captured the variation among samples. The stress value of 0.132 indicated that sample points within the same group exhibited heterogeneity (Figure 4B).

#### Microbial composition analysis

**Figure 5:**
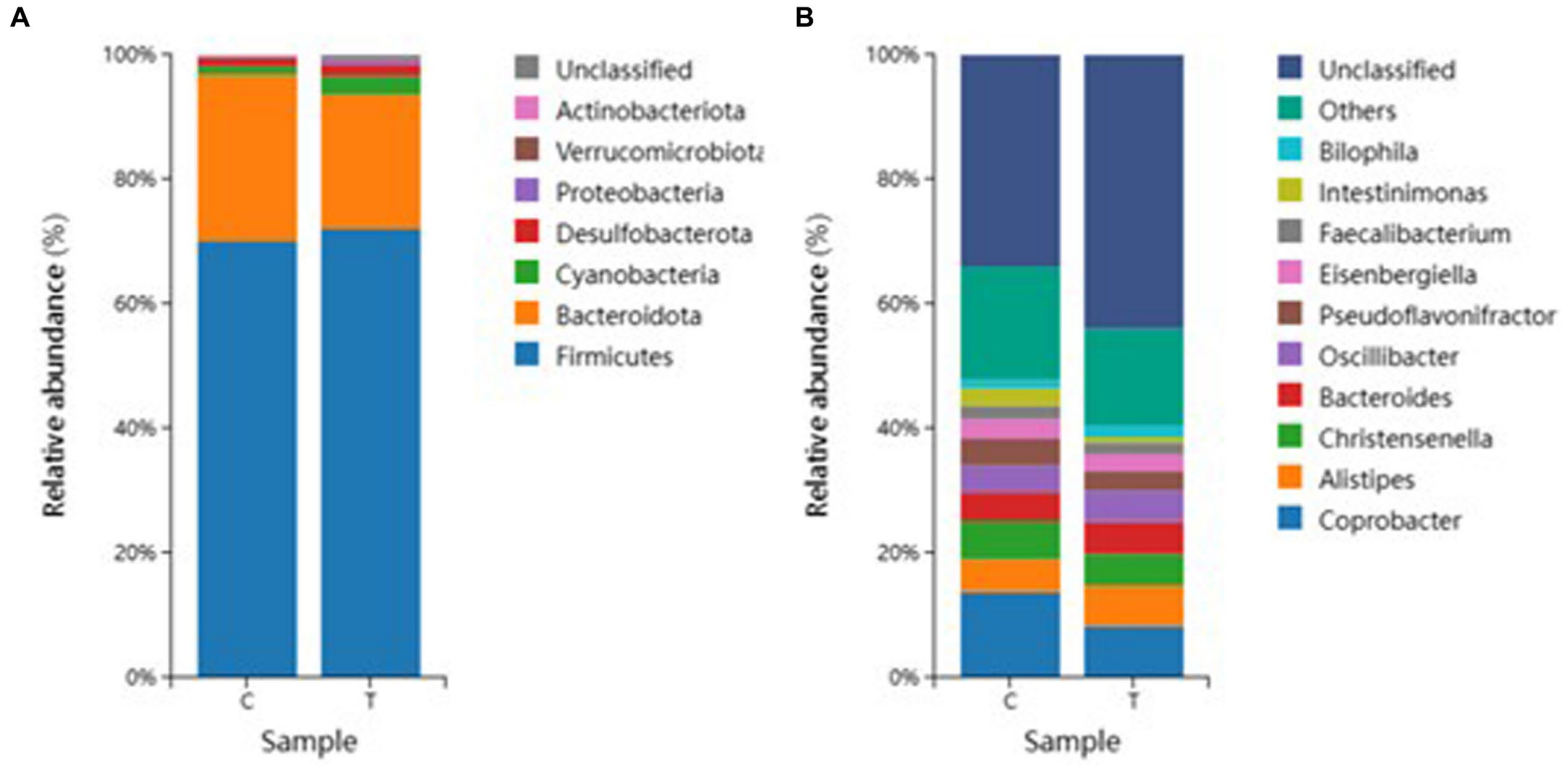
Species composition analysis. **(A)** Phylum level species composition and **(B)** Genus level species composition.

According to the species dilution results, we selected the top eight microorganisms with the highest abundance at the phylum level for relative histogram analysis (Figure 5A). Based on the statistical analysis of the species abundance of broiler intestinal microorganisms at the phylum level, we identified Firmicutes, Bacteroidetes, Actinobacteria, Cyanobacteria, Verrucomicrobiota, Desulfobacterota Proteobacteria, and un-known bacteria. Among them, Firmicutes accounted for more than 60% of the intestinal bacteria of broilers. The levels of Cyanobacteria in T-group broilers were higher than those in the C-group. The proportion of Bacteroidetes was about the same in both groups.

In a further analysis focused on the genus level, we selected the top twelve microorganisms with the highest abundance at the genus level in each test sample to make a relative histogram (Figure 5B). The analysis and statistics of the species abundance of broiler intestinal microorganisms at the genus level found *Coprobacter*, *Alistipes*, *Christensenella*, *Bacteroides*, *Oscillibacter*, *Pseudoflavonifractor*, *Eisenbergiella*, *Faccalibacterium*, *Intestinimonas*, *Bilophila*, and other unknown genera. The relative abundance of *Coprobacter* in the C group was higher than that in the T group. The distribution of *Intestinimonas* in group C was greater than that in group T, and the unknown bacteria and other bacteria in the C and T groups accounted for more than 60% of the total bacterial content.

## Discussion

The human health benefits of antioxidants, including their anticancer, antiviral, cardioprotective, and neuroprotective properties, are widely recognized (23–26). Cellular enzymes involved in oxidation and reduction play a vital role in counteracting oxidative damage caused by excessive production of reactive oxygen species (ROS). However, this cellular defence mechanism can be compromised due to disease or aging (27). Toona leaves contain natural antioxidants like flavonoids and polyphenols, which are important phenolic derivatives (28). Researchers, nutritionists, and food manufacturers are keen on understanding the health impact of dietary polyphenols. Polyphenolic compounds have a wide range of biological effects, including combating bacteria, reducing inflammation, protecting liver function, preventing blood clotting, and promoting vasodilation. These diverse functions are often linked to their scavenging of free radicals and antioxidant properties (29, 30). Antioxidants achieve their benefits through various mechanisms, such as reducing the concentration of molecular oxygen or oxidative metal ions, trapping and neutralizing ROS like O2− and hydrogen peroxide, scavenging free radicals that initiate chain reactions (e.g., •OH, RO• or ROO•), disrupting these chains, or quenching singlet oxygen (31). Free radicals are chemically reactive species with unpaired electrons in their outer orbitals, making them highly reactive and initiators of chain reactions (24, 32). Most free radicals in the body are ROS or reactive nitrogen species (RNS). To comprehensively understand the free radical scavenging properties of a substance, it’s recommended to use multiple assays. Accordingly, we selected one ROS (OH) and one RNS (DPPH) species to evaluate the free radical scavenging ability of TSE. Based on the *in vitro* antioxidant results of this study, although TSE has a lower free radical scavenging rate than VC, it also has certain free radical scavenging ability.

In the modern commercial breeding system, the elevated temperature environment has led to numerous adverse effects on the growth and development of poultry. Under high-temperature conditions, poultry’s feed intake decreases, growth rate slows down, intestinal health is compromised, oxidative stress intensifies, and the immune response is impeded (33). When the environment changes, animals adjust and restore their homeostatic conditions. In this process, certain stress responses are triggered (34). Abnormal temperature and rainfall patterns resulting from global warming pose various stressors on poultry farming, including technical, environmental, and nutritional challenges. These stressful conditions eventually result in oxidative stress (35). Research has demonstrated that oxidative stress can cause apoptosis in follicle cells, reducing the number of follicles, and consequently leading to diminished egg production (36). Moreover, stress can reduce egg quality characteristics, yolk lipid, and cholesterol con-tent. In the case of laying hens, sexual maturity and the onset of egg-laying are of great importance, and oxidative stress can adversely affect their performance (34). In the case of chicks, oxidative stress during growth and development may result in nutrient deficiencies that affect liver and bone metabolism (37). To mitigate the impact of oxidative stress in poultry farming, managers typically enhance poultry adaptability by improving feed nutrition and poultry housing, thus enhancing growth performance. The TSE selected in this study is a novel type of plant-based feed additive, which is less commonly used in livestock and poultry production. However, through the analysis of the chemical composition of Toona sinensis, it was discovered that its flavonoids are widely utilized in livestock and poultry farming, playing a significant role in enhancing animals’ growth performance (38–40). In this study, we show that TSE has a positive impact on the growth performance of broilers. TSE increased AWG and reduced the F/G ratio in chickens in the starter phase. There was also a decrease in the F/G ratio in chickens in the total period. Moreover, 1,200 g/t TSE was found significantly superior to VC and VE supplements. The TSE-mediated enhancement of broilers’ growth performance can be attributed to its flavonoids. They may help alleviate oxidative stress and regulate beneficial intestinal microflora. Good intestinal health is pivotal for animals’ digestion, ab-sorption, and immune functions (41–43). Furthermore, flavonoids can enhance animals’ blood circulation, increasing oxygen and nutrient supply (44, 45). Proper blood circulation is indispensable for enhancing an animal’s physical activity, growth rate, and overall growth performance. As for the reason that TSE is only effective in the starter phase and not effective in the finisher phase, we speculate that it may be because in the early stage of broiler growth (1–21 days), plant extracts may promote growth and improve feed utilization, thus significantly improving the production performance of broilers. However, as broilers grow to the later stage (22–42 days), there may be saturation of feed intake and slowing down of growth rate, resulting in the effect of plant extracts is no longer obvious, but the specific influencing factors still need to be further explored.

Enhancing poultry product quality and nutritional value is a key goal in the farming industry, responding to the growing demand for healthy food. Natural plant extracts are frequently added to poultry feed to optimize nutrient utilization. Toona sinensis have rich in nutrients and biologically active compounds (46). Our study revealed that dietary TSE significantly enhances the utilization of DM and OM in broilers, with the 1,200 g/t addition group showing the best results. Furthermore, TSE boosts broilers’ nitrogen utilization, reducing nitrogen emissions. However, TSE’s regulatory mechanisms on broiler nutrient efficiency remain underexplored. Analysis of TSE reveals that poly-phenolic compounds and cellulose stimulate intestinal peristalsis, digestive juice secretion, and nutrient absorption (47, 48). Flavonoids and phenolic acids maintain intestinal microbe balance, promoting beneficial bacteria growth while reducing harmful bacteria, thus enhancing intestinal health. This supports better nutrient absorption and utilization (49–51). Flavonoids and polyphenols also inhibit RNS production in urine while improving nitrogen absorption and utilization, ultimately reducing nitrogen excretion (52). In summary, TSE may reduce fecal nitrogen content and improve growth performance of broilers by inhibiting nitrogen excretion and promoting nitrogen utilization, but the specific regulatory mechanisms and details may require further research and discussion. Broiler farming has transitioned to large-scale production, driven by increased productivity and technological advancements. Current priorities involve optimizing broiler meat characteristics that influence consumer purchasing decisions, such as appearance, texture, juiciness, wateriness, firmness, tenderness, smell, and flavor. Additionally, quantifiable properties like water holding capacity, shear force, drip loss, cooked meat ratio, and pH are essential considerations (53). In the context of the global antibiotic ban, poultry nutrition plays a crucial role in poultry meat quality and safety. Finding feed additives that prevent poultry diseases and improve meat quality is a pressing issue in the poultry breeding industry. Our experiment reveals that incorporating TSE into broiler diets reduces chicken drip loss, enhances cooked meat ratios and pH, and extends shelf-life. However, TSE and antioxidants did not improve tenderness; instead, they increased meat shear force. Currently, there is limited research on TSE’s effect on broiler meat quality. The results showed that polyphenol extracts from eucalyptus leaves, flavonoids from scutellaria and oyster mushrooms had positive effects on animal meat quality (54–57). We find that antioxidant compounds in the TSE reduce lipid and protein oxidation in chicken meat, delaying oxidative aging and enhancing color, taste, and storage stability. This suggests that TSE’s antioxidants, such as polyphenols and flavonoids, inhibit lipid oxidation and radical reactions, extending chicken freshness and improving water retention and texture. However, shear force varies for different meats, making it an insufficient criterion for meat quality.

Antioxidant indices T-AOC, SOD, GSH-PX, and MDA in serum have different meanings for animals. These indexes can reflect the antioxidant capacity and oxidative stress state in animals. A higher level of T-AOC, an indicator of overall system capacity, indicates a stronger antioxidant capacity in the animal, which helps fight against free radical damage (58). SOD is an important antioxidant enzyme, and its activity level can reflect the animal’s ability to scavenge superoxide anions (59). GSH-PX can protect cells from oxidative damage by reducing hydrogen peroxide, and its activity level reflects the animal’s ability to cope with oxidative stress (60). MDA is a representative of lipid peroxidation products in cells, produced by lipid peroxidation reactions caused by oxidative stress, and the level of MDA reflects the degree of oxidative damage in animals (61). We showed that the addition of antioxidants and TSE in the diet improved the antioxidant capacity and significantly reduced the oxidative damage of broilers. Additionally, the antioxidant effect of the test group with TSE added at 1200 g/t was similar to that of the test group with VC and VE. The specific mechanism by which TSE enhances anti-oxidant capacity in animals was not considered in this study and needs further examination. However, polyphenols and flavonoids in plant extracts have been shown to have antioxidant activity and may positively affect the antioxidant capacity of animals (62, 63). Polyphenolic compounds are excellent hydrogen or electron donors with stable free radical intermediates, inhibiting the free radical chain reactions, and thereby reducing the occurrence of oxidation reactions (64). Flavonoids can donate hydrogen to lipid free radicals and transform themselves into stable phenolic free radicals, reducing the transmission rate of auto-oxidative chain reactions (55, 65). Furthermore, polyphenols and flavonoids can enhance the antioxidant capacity of cells by promoting the expression of antioxidant genes through the regulation of intracellular signaling pathways, such as the Nrf2/ARE signaling pathway (66, 67).

A healthy intestinal environment ensures the well-being of the animal. The number and types of microorganisms in different intestinal tracts of broilers vary significantly. The dynamic balance of intestinal microorganisms in broilers ensures normal growth performance, nutrient utilization metabolism, physiological and biochemical roles, immune system, and antioxidant processes. Intestinal microbes can be classified into beneficial and harmful bacteria (68). Under normal circumstances, intestinal microorganisms coordinate with host animals to ensure the dynamic balance of intestinal microorganisms. However, sudden changes in the external environment and alterations in animal feed can affect the animal’s body to some extent, leading to the proliferation of harmful bacteria and an excessive bacterial population, ultimately affecting the normal growth performance of animals (69). Therefore, maintaining the balance of intestinal flora plays a crucial role in the host’s health (70), making it an essential prerequisite for ensuring the normal physiological well-being of animals. Research has shown that Chinese herbal medicine monomers or plant extracts, and compound preparations can significantly enhance the structure of intestinal flora, increase the abundance of beneficial intestinal bacteria, and reduce the number of harmful bacteria, thus preventing and treating diseases (71). Currently, the research on plant’s impact on poultry intestinal microbes mainly focuses on extracts. It was reported that dietary supplementation of astragalus polysaccharides and glycyrrhizin polysaccharides increased the richness and diversity of microflora in the cecum of broilers (72). Additionally, studies have found that adding eleuthero and achyranthes knuckle extracts to the diet can increase the number of lactic acid bacteria in the intestinal tract of poultry and reduce the number of *Escherichia coli* and Salmonella (73, 74). However, there are few reports on the application of TSE in livestock and poultry production. Studies have shown that the microbiota of mammals is passed down vertically to the next generation, while poultry is the egg-laying animals, the microbial composition of poultry seldom influences the microbial characteristics of their offspring, resulting in a significant decrease in the diversity and abundance of intestinal flora in poultry (75). Upon hatching, changes occur in the poultry’s intestinal flora due to various internal and external factors that mainly affect digestion and absorption processes taking place in the small intestine (76). The ileum, situated after the jejunum and connected to the cecum, constitutes the final section of this organ system. Therefore, this study sought to investigate the impact of TSE on the microflora present in broilers’ ileums. The result showed that the inclusion of TSE had no impact on the gut microbial diversity of broilers. By analyzing and comparing the intestinal microbial communities, it was observed that the addition of TSE did not alter the composition or disrupt the homeostasis of the intestinal microbiota. Combining the growth performance and nutrient digestion and utilization data of this experiment, we have observed a positive impact on broiler growth performance and nutrient utilization with the addition of TSE. However, no significant changes were noted in the ileal microflora, this is consistent with the results of Shang et al. (77). This suggests that while gut flora is one factor influencing individual productivity, it cannot be solely relied upon for improving growth performance. Other relevant factors such as feed composition, feeding environment, and feeding management need to be considered.

## Conclusion

*In vitro* antioxidant results showed that TSE has better scavenging rates against DPPH and hydroxyl free radicals. During the stater phase, dietary supplementation 1,200 g/t TSE significantly decreased the AFI, F/G, and F/G was also decreased during the whole feeding phase. In addition, adding 1,200 g/t TSE to the diet increased the nutrient utilization, meat quality, and serum antioxidant function of broilers.

## Data availability statement

The original contributions presented in the study are included in the article/supplementary material, further inquiries can be directed to the corresponding author.

## Ethics statement

The animal studies were approved by Faculty Animal Policy and Welfare Committee of Gansu Agricultural University (No. GSAU-Eth-AST-2021-001). The studies were conducted in accordance with the local legislation and institutional requirements. Written informed consent was obtained from the owners for the participation of their animals in this study.

## Author contributions

XZ: Conceptualization, Data curation, Formal analysis, Software, Visualization, Writing – original draft, Writing – review & editing, Investigation. BD: Data curation, Supervision, Writing – review & editing. MW: Data curation, Writing – review & editing. JL: Data curation, Writing – review & editing. SQ: Data curation, Writing – review & editing. FN: Data curation, Writing – review & editing. DT: Conceptualization, Data curation, Funding acquisition, Project administration, Resources, Validation, Writing – review & editing.
